# A Peep at Russia and Home by Sweden

**Published:** 1891-10-17

**Authors:** 


### SHORT TRIPS FOR SHORT HOLIDAYS.

### II.—A PEEP AT RUSSIA AND HOME BY SWEDEN.

It is a long cry from England to Russia. A visit to that country had been many a time talked about, and as often postponed, until one Saturday evening in August we stepped on board a prehistoric steamer which plies between Hull and St. Petersburg. The North Sea kept up its reputation—it was decidedly rough.

Once in the Skagerrack things improved much for us and by the time we rounded the Skaw all was contentment and enjoyment. The misery of the two preceding days was forgotten, as if it had never existed. The sail on the Kattegat was charming. Beautiful was the sea to look on. Only the faintest ripple stirred its surface, and when night approached the scene became more lovely still. The sun would set in a way which he never seems to do elsewhere than at sea—amid gold and vermilion and pale blues and greens, at last blending all colours together in a manner that not the greatest painter could pourtray on canvas. Twilight remained long with us, as if it were loth to go. Onwards the vessel sped, passing close to Elsinore and Copenhagen. A little further up the Sound we had one of those experiences which an ocean voyage could never give. The fleet of timber-laden vessels from the Baltic ports had lain weather-bound, but lately the wind had changed, and now every one was on the wing, wending its way to some far-off haven. It was just as darkness was gathering around us, and in the narrowest part of the Channel we met the most of these vessels. They were of almost every kind, from the pretty fore and aft schooner to the stately ship with her canvas piled from the deck to the royal yards. We had them on the port side, so that each one showed us the red light. They went slowly past in one continuous stream. It seemed as if an army of giant torch-bearers had been sent out to light our way across that summer sea. One hundred were counted—two hundred—three hundred—and then the reckoning was lost.

On leaving our cabin on the seventh morning from Hull, we found the steamer had anchored close to Cronstadt. Presently we were boarded by the Customs officers and by the police. Every passport was examined, and the name compared with other names in a list held by the police. The hatches of the vessel were sealed, and a number was placed on each portmanteau and box or bag, after which nothing must be interfered with by the owners. The police and interpreter left us, but a Customs officer (who, in England, would have been mistaken for a crossing-sweeper, save that he had a sword slung over his shoulder) was left in charge of our luggage until we arrived in St. Petersburg, where everything was turned out and laid in line numerically in front of the Custom House. Here a thorough search was made, much time wasted, and a good stock of patience needed by those whose numbers were high.

Viewed from the water, St. Petersburg is rather striking. The large gilt dome of St. Isaac's is the most prominent object, and it can be seen afar off with its cross towering over all. Close to it is the long, thin spire of the Admiralty, and on the opposite side of the river is a similar spire (both are gilt), that of the church of St. Peter and St. Paul.

There are many good hotels in St. Petersburg, and in three or four of the best of them the tourist will find at least one official who can speak English. As regards language, English, French, and German are about equally useful in St. Petersburg, but in Moscow German will carry one further than the other two.

St. Petersburg is disappointing in many ways. We walk along the streets, termed prospects and oulitzas, and, although many of them are of great length and very wide, they strike us as being bare and unfinished. We notice, too, that scarcely one of the houses, even in the Nevskoi Prospect, is what it appears to be. There is nothing but stucco. The squares are of enormous area, but, apart from the statues sometimes seen in the centre, the eye hardly ever lights on anything that pleases. On the right of the Nevskoi is one of these squares with a statue of Catherine II. It was near this that the beautiful Madame Lapoukyn met with her terrible punishment during the reign of the Empress Elizabeth. An involuntary shudder passes over us, and then memory conjures up another "noble white vision" who ascended the scaffold in Paris a generation later. How like the despotism of the one country was to the liberty of the other!

There is scarcely a decent piece of architecture to be seen anywhere. The streets are mere repetitions of each other. One, indeed, has a long colonnade—an insipid imitation of the insipid Rue de Rivoli. Miskewicks says that human hands built Rome, Divine hands created Venice, but St. Petersburg is the work of the devil. The Taurida Palace is said to be fairly characteristic of the great houses. "The marble is all false; the silver is plated copper; the pillars and statues are of brick, and the pictures are copies."

Many of the residences of the nobility are now built on the broad, fine quays which face the Neva, as the great people have been driven from the Nevskoi by the inroad of shops. Along the quays one occasionally sees a striving after better things—real stone makes its appearance, at least some of the houses are partially built of it.

In St. Petersburg there are, however, many grand things to see. Perhaps the best is the Hermitage. Here there is a truly splendid collection of pictures. Almost every school is represented, and represented by many works of many artists. Here, too, are several articles made by Peter the Great himself, and interesting for that reason if for no other. There is also a fine collection of antiquities and of modern articles. Exquisite mosaics vie with each other to carry off the palm. Huge vases—valuable for their size only, one feels inclined to say—jewelled boxes, swords covered with emeralds and diamonds, and saddle-cloths embroidered with precious stones! Room after room is filled in the richest profusion with all manner of objects, until at last the eye wearies with looking. It can take in no more. Close by the Hermitage is the Winter Palace. An order from the Embassy is required to enable us to visit this; and when the order has been obtained, it must be left in the Palace until next day, when the visitor will almost certainly be admitted. The Hermitage and the Winter Palace are supposed to be free; but "tipping" has been in Russia raised to one of the fine arts, and it takes about five roubles to get through the Winter Palace alone. Then there is the Museum of Imperial carriages. We see rows of carriages gilt completely over—others have their panels beautifully painted; in one instance at least by Watteau. Some are adorned with diamonds and rubies, but the most of the "jewels" seen here and on the harness are "shams"—Siberian crystal or something of that kind. In a corner stands the plain brougham occupied by Alexander II, on that day when he was assassinated—the panels are rent and torn by the bomb—the first one thrown at the Czar. There is much fine tapestry on the walls of this Museum.

The Foundling Hospital is wonderful of its kind and will be visited by everyone interested in such institutions. Those who have time will not neglect Peterhoff, the Versailles of Russia; nor will they omit a sail up the Neva and a drive round the Islands. The young tourist will probably go to the "Zoo" where he will see barely enough quadrupeds to excuse the name given to the place.

In his first walk up the Nevskoi the visitor cannot fail to see the Kazan Cathedral, and he will hardly know whether to laugh or fly into a rage as he looks on this ambitious, sham imitation of St. Peter's. On entering it, however, he will cease to think of the exterior as he wanders amid the stately columns or gazes on the richly frescoed walls, or has his attention rivetted by the dazzling Iconostos with its gates of silver and pillars and rails of the same metal. He will see the picture of our Lady of Kazan adorned with jewels of immense value. He will be struck, too, with the worshippers and with the service, if one be going on. He will see the people crossing themselves; falling on their knees and touching the floor with their foreheads, and he will hear perfect singing by the choir and exquisite intoning by the priests, as clad in their gorgeous vestments they take the solo parts of the service.

Still more wonderful is the Cathedral of St. Isaac. As the Kazan is an imitation of St. Peter's, so is St. Isaac's a sort of poor copy of St. Paul's. Its proportions and general arrangement have been severely, and we think justly, criticised; but many of the parts are beautiful in themselves, and it is unquestionably the finest building in St. Petersburg. It is no a sham. The walls are of stone. The columns supporting the pediments are most lovely. Their bases and capitals are of bronze, and their shafts are monoliths of Finland granite. So are the steps leading to the various entrances, and the granite blocks are here of enormous size. Inside it is similar to the Kazan. Indeed, the Russian churches, being all built in the form of a Greek cross necessarily gives to the interiors a strong family likeness. In the church of St. Peter and St. Paul we find the tombs of the Czars since Peter the Great. They are of white marble.

The almost universal way of getting about St. Petersburg is the droski. This carriage is simply a small victoria on very small wheels. Many of them have no hoods, and there is then scarcely any side or back to the seat, so that one might easily fall out as the vehicle whirls round a corner or jolts over the frightful, uneven pavements. To prevent accidents it is by no means uncommon to see a man with his arm around the waist of his lady friend—a custom which might be pleasant, or otherwise, according to circumstances. The driver is clad in a padded coat of blue cloth reaching to his feet, and confined at the waist by a belt of some pretty figured material. His head gear closely resembles a tall hat which had collapsed from some one sitting on it. He holds a rein in each hand, uses no whip: simply speaks to his horse, and the moment you are seated the animal bounds off like a ball from a catapult. Streets accidents are said to be very numerous, and we can readily credit the statement. There are no fixed charges, and a bargain has to be made every time with the driver, who generally takes one-third of what he asks. As the "fare" gets in, the driver says something, which Hare translates as "Hold on in God's name, little father."

When one remembers that St. Petersburg now contains nearly one million of inhabitants; that the oldest part is not yet two hundred years old, and that much of it is very much less than that age, it will have to be admitted that it is a wonderful city. And yet the visitor will be almost sure to agree with Hare when he says, "the same impression, probably, is left on the minds of all who have visited St. Petersburg—a prevailing sense of the vastness of everything: the squares, the streets: the palaces: the overgrown, desolate suburbs—and in spite of the interest of much that is curious and strange—a weariness of a city so beautiless, so uncouth, and so irksome to a stranger in the bondage of its petty restraints."

*(To be concluded.)*

